# Dynamically sculpturing plasmonic vortices: from integer to fractional orbital angular momentum

**DOI:** 10.1038/srep36269

**Published:** 2016-11-04

**Authors:** Yu Wang, Peng Zhao, Xue Feng, Yuntao Xu, Fang Liu, Kaiyu Cui, Wei Zhang, Yidong Huang

**Affiliations:** 1 Department of Electronic Engineering, Tsinghua National Laboratory for Information Science and Technology, Tsinghua University, Beijing, China

## Abstract

As a fundamental tool for light-matter interactions, plasmonic vortex (PV) is extremely useful due to the unique near field property. However, it is a pity that, up to now, the orbital angular momentum (OAM) carried by PVs could not be dynamically and continuously tuned in practice as well as the properties of fractional PVs are still not well investigated. By comparing with two previously reported methods, it is suggested that our proposal of utilizing the propagation induced radial phase gradient of incident Laguerre-Gaussian (LG) beam is a promising candidate to sculpture PVs from integer to fractional OAM dynamically. Consequently, the preset OAM of PVs could have four composing parts: the incident spin and orbital angular momentum, the geometric contribution of chiral plasmonic structure, and the radial phase gradient dependent contribution. Moreover, an analytical expression for the fractional PV is derived as a linear superposition of infinite numbers of integer PVs described by Bessel function of the first kind. It is also shown that the actual mean OAM of a fractional PV would deviate from the preset value, which is similar with previous results for spatial fractional optical vortices.

Light beams with spiral phase front can carry quantized orbital angular momentum (OAM)^1^,^2^, which are also known as optical vortices^3^. Due to various distinguishable characteristics, OAM has widely expanded the applications of both quantum and classic optics^4^,^5^, imaging^6^, sensing^7^, micro particle manipulation^8^,^9^, *etc*^10^. In recent years, optical vortices with fractional OAM are further investigated to fully explore some additional uniqueness, such as the radial opening on intensity pattern and mode decomposition into infinite numbers of Laguerre-Gaussian (LG) modes^11^. Based on those attributes, some fascinating applications of fractional OAM, including micro particles transportation guiding^12^, nearly perfect realization of the famous Hilbert’s Hotel paradox^13^ and high dimensional quantum entanglement of two photons^14^ have been demonstrated.

As a combination of OAM and plasmonics, chiral plasmonic structures for plasmonic vortices (PVs) are under active investigations nowadays^15^,^16^. Owning to the near field properties of plasmonics^17^, PVs are quite suitable for manipulating light-matter interactions, revealing many fundamental phenomena and applications, such as optical lattice formation^18^, spin-Hall effect^19^, spin symmetry breaking^20^, polarization analyzing^21^ and micro particle rotating/trapping^22^. In addition to integer PVs, fractional PVs have also been analytically investigated recently^23^,^24^. However, previously proposed methods^23^,^24^ for sculpturing fractional PVs cannot be dynamically tunable in practice. Besides, it is still lack of an explicit analytical description for fractional PVs until now and the actual mean OAM of a fractional PV is still unknown, while the spatial optical vortices with fractional OAM have been clearly investigated already^25^,^26^,^27^.

In this paper, by comparing with two previously reported methods, *i.e.*, modifying the chiral plasmonic structures^23^,^24^ or tuning the freespace wavelength *λ* of incident LG beam^23^, it is suggested that our proposed method of utilizing the propagation induced radial phase gradient of LG beam is a promising candidate to sculpture PVs from integer to fractional OAM dynamically. Specifically, spherical wave like behavior of LG beam would induce radially gradient to phase profile during propagation^1^,^28^. Once the plasmonic excitation plane is not right on the optical waist of incident LG beam, this radial phase gradient would break the implied radial homogeneity of chiral plasmonic structures, which in turn, introduces an additional contribution to the OAM of excited PVs. The modified expression for the preset OAM of PVs is derived to include this contribution, which could be either an integer or a fraction, together with the incident spin angular momentum (SAM), OAM and the geometric contribution of chiral plasmonic structure. Moreover, we have analytically derived the explicit expression for fractional PVs, which is actually a linear superposition of infinite number of integer PVs. The relation between the actual mean OAM and the corresponding preset value of a fractional PV is then investigated, which shows similarities with results of spatial optical vortices with fractional OAM^25^,^26^,^27^. Both analytical and numerical results are illustrated to validate our proposal and we believe this work would provide a flexible sculpturing method for PVs with continuously tunable OAM from integer to fraction, by just properly settling the incident LG beam parameters without changing the carried topological charge.

## Results

### Basic theory of integer PVs

**Figure 1:**
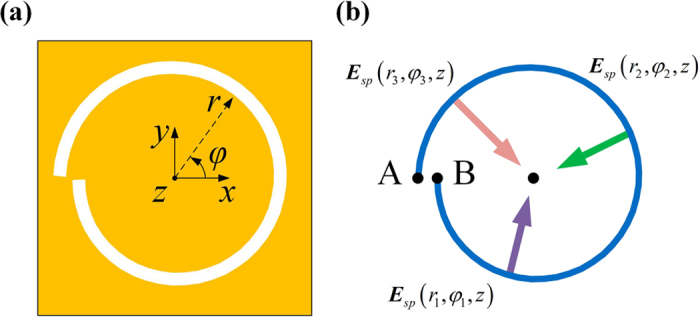
The schematic diagram of ASG and excitation of PVs. (**a**) The coordinate of the ASG in this work and (**b**) the diagrammatic sketch of the principle for generating PVs.

First of all, we will briefly review the basic theory of generating PVs with integer OAM. As shown in Fig. 1(a), the Archimedes’ spiral grooves (ASG) is typically considered as the chiral plasmonic structure to introduce a geometric contribution, whose groove trajectory is given by


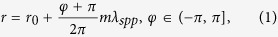


where *λ*_*spp*_ is the wavelength of excited surface plasmonic wave, *r*_0_ is the minimum radius of the ASG, *φ* is the azimuthal angle and *m* ∈ *Z* is the quantized geometric contribution. A LG beam with circular polarization is incident from the backside and the complex amplitude in the polar basis (*r, φ*) is given by^29^


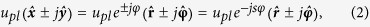


where *s* = ±1 stands for the quantized SAM per photon (representing right-handed and left-handed circular polarization, respectively). The term of *u*_*pl*_ is the complex amplitude of LG beam under paraxial approximation^1^:


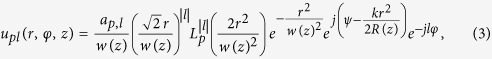


where *p* is the radial index and *l* is the topological charge. *α*_*p,l*_, *w*(z), 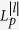 and *R*(*z*) donate the normalized amplitude coefficient, the beam width, the associated Laguerre polynomial, and the radius of wavefront curvature, respectively. And 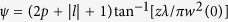 is the Gouy phase.

As inherently transversal-magnetic (TM) polarization, surface plasmonic waves could only be excited with the radially polarized component of incident electric field since it is perpendicular to the groove^30^. For any observation point (*R, ϕ, z*) near the PV center, the contribution from a certain point (*r, φ, z*) on the groove is given by^24^,^31^


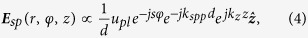


where 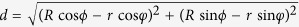 is the distance between the observation point (*R, ϕ, z*) and the excitation point (*r, φ, z*) on the groove, *k*_*spp*_ is the propagation wave number and *k*_*z*_ is a complex number depicting the intensity decaying in the *z* direction. Following the principle of Huygens’ integral^31^, the electric field at the observation point (*R, ϕ, z*) is given by


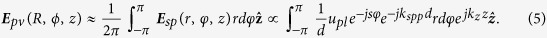


As the observation point is near the PV center, it is assumed that *R* << *r* so that 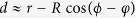 and 1/*d* ≈ 1/*r*. Furthermore, the propagation loss of plasmonics and the imbalance of LG beam intensity along the groove are ignored so that only LG beam’s phase profile matters. Equation (5) is then simplified as


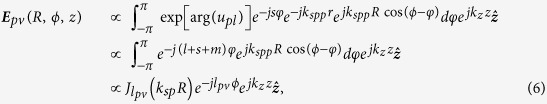


where *J*_*l*_ is the *l*-order Bessel function of the first kind and the OAM charge of PV is


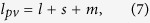


which consists of three independent parts: incident SAM, OAM and the geometric contribution of the ASG. This is the most common situation for PVs, *i.e.*, with controlled integer OAM.

### Methods for sculpturing fractional PVs: a comparison

Following equation (7), it is not hard to conclude that the phase variation along the ASG is given by


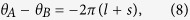


where the locations of point A and B are illustrated in Fig. 1(b). To generate integer PVs, the phase variation along the ASG should be an integer multiple of 2*π* due to the fact that both SAM and OAM numbers for any circularized LG beam are integers. Thus, *m* in equation (7), which is the geometric contribution of the ASG, should also be an integer and the distance between point A and B in Fig. 1 should be an integer multiple of *λ*_*spp*_. As *λ*_*spp*_ is directly related to the incident LG beam’s freespace wavelength *λ*^17^, this could be ensured by fixing the incident *λ* as a constant. Last but not the least, the structure of the ASG strongly implies a radial homogeneity of phase, *i.e.*, the phase profile of incident LG beam as an excitation should be radially invariant. In practice, the optical waist of incident LG beam is always aligned right on the ASG. Otherwise the propagation induced radial phase gradient of LG beam would break the implied radial homogeneity of the ASG.

If any one of the three prerequisites for generating integer PVs breaks, the phase variation along the ASG would not be an integer multiple of 2*π* anymore and fractional PVs would be excited as a result. Thus, there are three corresponding methods for sculpturing fractional PVs with employing LG mode as incident beam: modifying the structure of the ASG, tuning the freespace wavelength *λ*, and utilizing the radial phase gradient of LG beam. The fractional part is donated by *α* and the expression for the preset OAM charge is then updated as


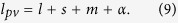


Modifying the structure of the ASG is quite intuitive and has already been investigated by some groups before^23^,^24^. With fixed incident *λ*, if the distance between point A and B in Fig. 1 is adjusted to be any fractional number multiple of *λ*_*spp*_, a fractional phase step and a corresponding fractional PV would be introduced. However, this method is hard to achieve dynamically controlling, as the fractional number *α* in equation (9) is completely fixed for a fabricated ASG structure. Consequently, a fractional number series is correspondingly determined. For example, the series is *l*_*pv*_ ∈ {…, −1.3, 0.3, 1.3, 2.3, …} when *α* = 0.3. For a given ASG, the fractional OAM *l*_*pv*_ could be only valued from one series (with varied incident SAM and OAM) and could not be any other fractional number.

Tuning the operating wavelength *λ* is also intuitive^23^. When the operating wavelength deviates from the designed one, *λ*_*spp*_ would be changed to *λ*_*spp*_ in response. Thus, the equivalent value of integer *m* is now a fractional number as 
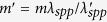
 and a fractional PV would be excited. However, this method is also inconvenient as generating LG beam is usually wavelength sensitive^10^. As an example, let us consider the spatial light modulator (SLM), which is the most commercialized optical unit for phase modulation. Once *λ* is changed, the SLM has to be adjusted according to its wavelength-phase response curve, which is highly time-consuming. Furthermore, the operating wavelength range for a certain SLM is usually limited so that a desired wavelength may not be achieved. Meanwhile, tuning the OAM of a PV is inevitably accompanied with varying the plasmonic wavelength, which should be avoided in real applications.

Utilizing the radial phase gradient of LG beam seems to be the most flexible option, which would be introduced in detail in the following, as the beam parameters can be readily controlled by employing appropriate *ABCD* matrices^31^. For LG beam involved in this work, only suitable optical lenses are required^32^. Fortunately, the transmission function of an arbitrary optical lens can be fully implemented on an SLM and is compatible with originally designed hologram pattern of SLM^33^, suggesting that an SLM could generate and focus a LG beam at the same time. This flexible method for sculpturing fractional PVs is dynamic and the response time is only limited by the refreshing frequency of the SLM (usually ~ 50 Hz).

### Sculpturing fractional PVs by utilizing the radial phase gradient of LG beam

Following equation (3), the phase profile of a LG beam is


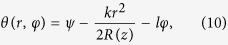


where the first term is the Gouy phase as mentioned, the second is the paraxial approximated radial phase gradient due to the spherical wave like beam propagation and the third characterizes the spiral phase front with corresponding OAM. It should be noticed that only the first term is homogeneous in a certain transverse plane (*z* is constant). The second is radially dependent while the third is azimuthally dependent.

**Figure 2:**
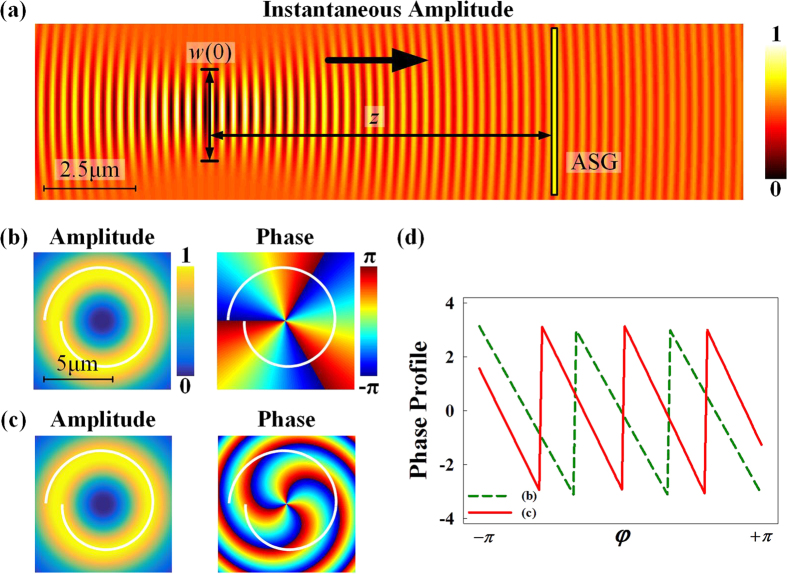
Sculpturing fractional PVs by utilizing the radial phase gradient of LG beam. (**a**) The lateral view of the instantaneous amplitude profile of a LG beam (*l* = 0) illuminating on the ASG, clearly showing the propagation induced radial phase gradient. The amplitude and phase profiles of two incident *LG*_03_ beam with parameters of (**b**) *w*(0) = 2.8 μm, *z* = 0 μm and (**c**) *w*(0) = 1 μm, *z* = 13 μm. (**d**) The phase variation profiles along the ASG.

Figure 2(a) shows the lateral view of the instantaneous amplitude profile of a LG_00_ beam (*w*(0) = 1 μm, *λ* = 633 nm) illuminating on the ASG. One could see the radial phase gradient clearly in the figure, as the radius of phase curvature *R*(*z*) is changing during the propagation. When the optical waist is right on the ASG (*z* = 0 and *R*(0) = ∞), the second term in equation (15) vanishes away, which is the integer case expressed by equation (7).

Figure 2(b) shows a specific example with LG_03_ mode (*w*(0) = 2.8 μm and *z* = 0 μm). The white curve is a projection of the ASG (*r*_0_ = 3μm, *m* = 2) onto the amplitude and phase profiles of incident LG beam. The beam parameters are designed to ensure that the amplitude profile is properly overlapped with the groove so that the assumption of ignoring intensity imbalance in equation (6) holds.

However, if the optical waist is not right on the ASG, the second term in equation (10) would introduce a radial phase gradient. For comparison, Fig. 2(c) shows an example of *w*(0) = 1 μm and *z* = 13 μm. As one could see by comparing with Fig. 2(b), there is an obvious fractional phase step between point A and B in Fig. 2(c). The different evolutions of phase profiles along the ASG for these two examples are shown in Fig. 2(d). For the example in Fig. 2(b) (dashed line), there are three complete phase periods from −*π* to *π*, while number of phase periods is between 3 and 4 as a fraction for the example in Fig. 2(c) (solid line).

The fractional phase step when utilizing the radial phase gradient of LG beam is derived as


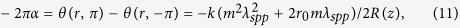


where the expression for the fractional number *α* is


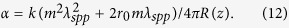


A radial phase gradient dependent contribution to OAM of fractional PVs is therefore introduced. Obviously, equation (9) would be degenerated to equation (7) when *z* = 0 as *α* = 0. But the most important thing is that, it inspires a flexible method to sculpture PVs with OAM from integer to a fractional number with constant *m* and *λ*_*spp*_, just by properly utilizing the radial phase gradient of incident LG beam. Thus, it is not necessary to vary the carried topological charge and the wavelength of incident beam as well as the ASG structure.

It is natural to ask where the additional OAM *α* comes from. In our proposal, the ASG does not take a closed loop and would recognize an additional phase step as shown in Fig. 2(c). As the OAM of LG beam is usually given by integrating the phase gradient along a path around the singularity (thus the number of phase variation from 0 to 2*π*)^34^, it could be found that due to the additional phase step, the open loop instead of the closed one is *effectively* utilized to recognize a different OAM of LG beam. However, this is not rigorous as: (1) such additional OAM contribution is only meaningful when considering the plasmonic excitation with ASG so that it is a valid concept for PVs but not for the LG beam; (2) the OAM charge of a LG beam should be consistent as *l* without any doubt, whatever the way of utilizing it or the value of *z*; (3) the method of integrating the phase gradient to identify the carried OAM of LG beam only holds when a closed loop is chosen. Consequently, the additional charge of *α* could not be viewed as the contribution of the carried OAM of incident LG beam since the carried OAM is implied by the azimuthal phase gradient but the fractional term of *α* relies on the contribution of the radial phase gradient of incident beam.

In order to quantitatively illustrate this matter of fact, a direct method is presented to obtain the fractional OAM with LG beam. In deriving equation (6), we have made the assumption that only the phase item of LG beam is considered. It is still valid even when away from the optical waist as the amplitude profile is properly overlapped with the groove as shown in Fig. 2(c). With equation (6) and equation (10), the PV is therefore given as


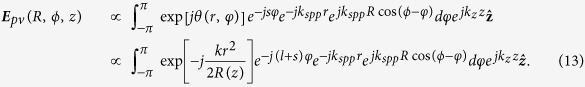


Note that an additional radially dependent item 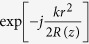 is introduced compared with equation (6). This item would vanish when at the optical waist of LG beam. After ignoring the constants, it is not hard to find that


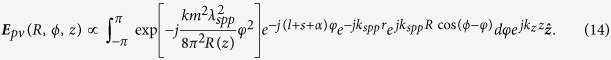


One could see that *α* is introduced by the additional radially dependent item. Furthermore, the exponential of high order angular item *φ*^2^ is ignored, as it is small compared to the dominant exponential of item *φ*, which characterizes the OAM property of a beam. Intuitively, the linear relation between phase and *φ* is also obvious in Fig. 2(d). Finally, we could obtain the expression:


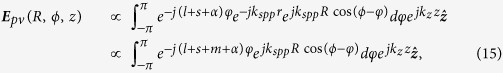


where 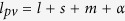 is obtained.

By using the principle of Huygens’ integral, the phase of incident LG beam at each point on the groove contributes to the formation of PVs. When away from the optical waist, the additional radially dependent phase item emerges and should be recognized together with the azimuthally dependent phase item *e*^−*jlφ*^. From the above derivation, it is obvious that *α* is separately and independently recognized from the radial phase gradient of LG beam. It should be mentioned that the OAM contribution induced by the azimuthal phase gradient of LG beam is still *l*, without any doubt.

Above all, the additional fractional OAM *α* comes from the radial phase gradient of LG beam (though it would not introduce any additional OAM contribution to LG beam itself), compared with *l* that comes from the azimuthal phase gradient of LG beam.

### Properties of fractional PVs

To further explore the properties of fractional PVs, it is important to realize that equation (9) *does not* simply suggest that we could rudely conclude fractional PVs being described by 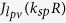, in which *l*_*pv*_ is now a fractional number. Analytically speaking, it is not true as the derivation of equation (6) involves the integral identify of Bessel function, which *does not* hold for a fractional number. Furthermore, a fractional optical vortex typically involved radial openings on intensity pattern, *i.e.*, azimuthal discontinuities^11^, equation (6) cannot depict this matter of fact as its intensity is azimuthally independent. Thus the field expression for fractional PVs should be further developed.

Following equation (6), we could first obtain the field expression of an arbitrary PV as


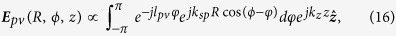


where *l*_*pv*_ is integer or fractional. With Fourier expansion, both the integer and fractional phase item could be simply decomposed as


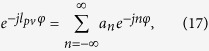


with the coefficient of


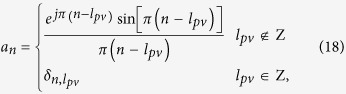


where *δ*_*i,j*_ is the Kronecker delta function. Therefore, the fractional PVs could be described by


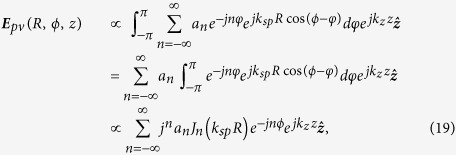


Equation (19) illustrates the fact that a fractional PV is actually a linear superposition of infinite numbers of integer PVs. In additional to the coefficient *a*_*n*_, one should pay attention to the additional phase shifts *j*^*n*^ for different integer PVs, resulted by employing the integral identify of Bessel function. The actual mean OAM of a fractional PV can be further expressed as


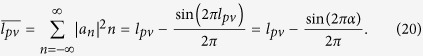


It is intriguing to see that the actual mean OAM of a fractional PV is *not* equal to the preset value unless the preset value is an integer (*α* = 0) or a half integer (*α* = 0.5). In scopes of spatial optical vortices, similar results were first reported by Berry^25^, then experimentally verified by Leach *et al*.^26^ and finally described with quantum mechanics by Götte *et al*.^27^. It is not surprising that the previous results are similar with equation (20), as both the spatial optical vortices and PVs could be described by the same phase item e^−*jlφ*^ and the phase shift terms of *j*^*n*^ in PVs do not affect the contribution of each integer PV on the final OAM.

### Numerical simulations

**Figure 3:**
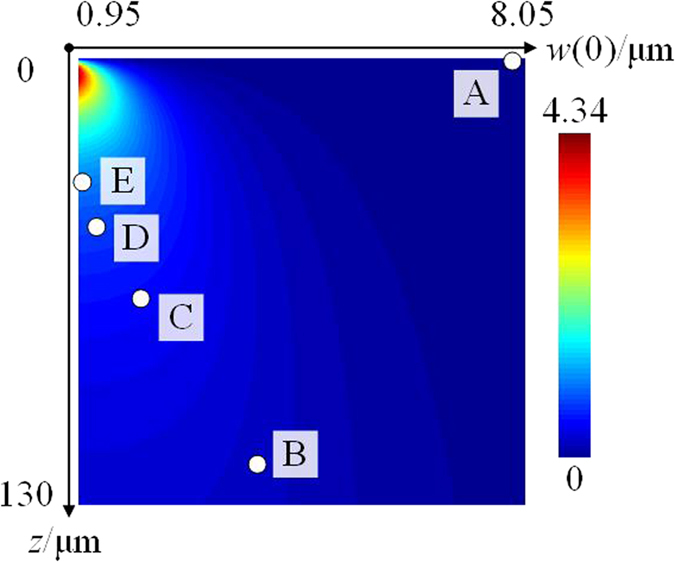
The analytically calculated results of *α* versus varied optical waist *w*(0) and propagation distance *z*. The Archimedes spiral groove is fixed as *r*_0_ = 9 μm and *m* = 4. Points A ~ E correspond to different combinations of LG beam parameters, as five concrete cases.

Based on the abovementioned properties of fractional PVs, finite-difference time-domain (FDTD) numerical simulations have been carried out to verify our proposal. The ASG is fixed with *r*_0_ = 9 μm and *m* = 4 as an example. Figure 3 shows that the analytically calculated *α* versus varied optical waist *w*(0) and propagation distance *z*. One should notice that the paraxial approximation condition requires the divergence angle small enough, which is 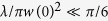 with *l* = 0^31^. Therefore, the lower limit for *w*(0) is chosen to be ~1 μm as *λ* = 633 nm in this work. In principle, *α* can be continuously varied 0 to 4.34 by varying the two LG beam parameters of *w*(0) and *z*. However, the amplitude profile should be settled to achieve an appropriate overlap with the groove (similar as shown in Fig. 2) so that the assumption of ignoring intensity imbalance in equation (13) would hold. Therefore, the optical waist has to be carefully chosen.

To demonstrate the tunability, five concrete cases (point A~E in Fig. 3) with different combinations of *w*(0) and *z* have been calculated with constant values of *l* = −3, *p* = 0 and *s* = 1 (right-handed circular polarization). The calculating parameters of five cases are listed in Method section. According to equation (9), the expression of *l*_*pv*_ for both integer and fractional PVs would be:


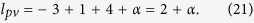


**Figure 4:**
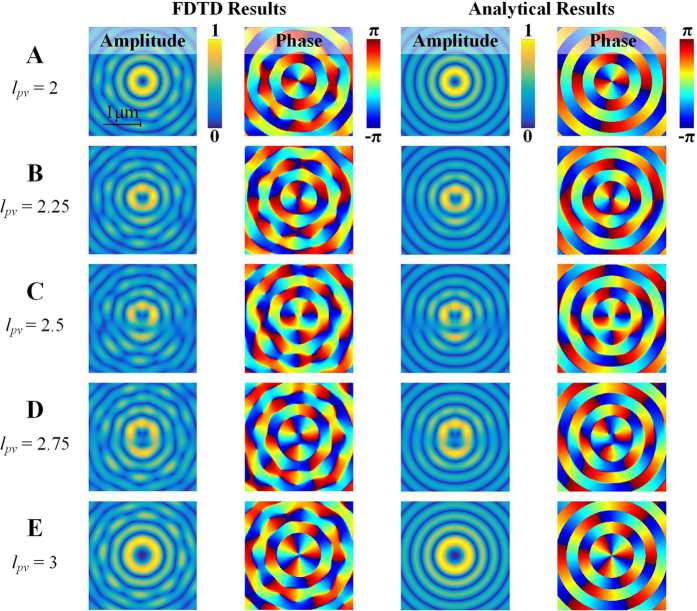
The OAM charge (*l*_*pv*_) of PVs is continuously tunable from 2 to 3, as either an integer or a fraction, by varying the radial phase gradient dependent contribution *α*. Both FDTD and analytical results of the five cases in Fig. 3 are shown for comparisons.

The FDTD result is shown in Fig. 4. For case A, the optical waist is right on the ASG (z = 0). Both the annual amplitude profile and the perfect anti-clock phase variation of *π* ~ −*π* for two times indicate that *l*_*pv*_ = 2 as *α* = 0. Furthermore, *α* is varied from 0.25, 0.5 to 0.75, 1 for case B~E with the optical waist away from the ASG. Compared to case A and E, the splitting of phase singularities in case B~D does indicate that the fractional OAM is successfully generated^11^. Furthermore, as an additional phase singularity is gradually moving towards the center with increasing *α*, the amplitude profile is also distorted. With increasing *α*, there is an amplitude gap (radial opening) that breaks the integrity of the originally annual profile (case A~C) at first. Then the intensity pattern would gradually develop back to an annual profile but with a larger radius (case C~E).

The FDTD results are compared with analytical ones from equation (19) in Fig. 4. They match each other quite well while ignoring the small calculation errors from FDTD simulation. It is worth to mention that the analytical derivation of fractional PVs does not rely on the employed sculpturing method so that equation (19) would hold for any fractional PV in spite of the implementation method. Such agreement indicates the fact that sculpturing fractional PVs by utilizing the radial phase gradient of LG beam is analytically reasonable and justifiable.

In order to further validate the fractional properties of PVs, mode decomposition into an orthogonal basis has also been carried out. The amplitudes of different composing components (integer PVs) of a fractional PV could be numerically calculated as


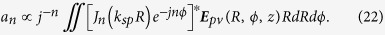


**Figure 5:**
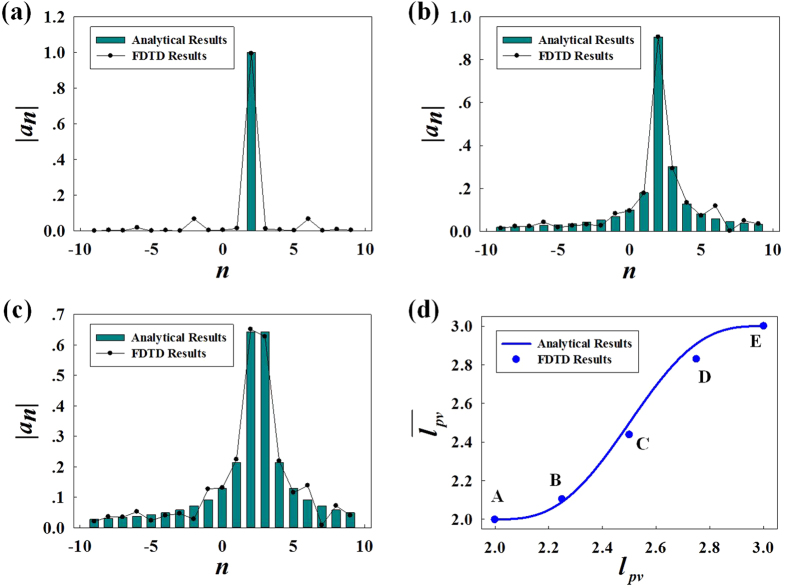
Comparisons between analytical and FDTD results. (**a**–**c**) Comparisons of |*a*_*n*_| with *l*_*pv*_ = 2, 2.25 and 2.5, respectively. (**d**) The calculated actual mean OAM (dots) from FDTD compared with the analytical predictions (solid line).

In Fig. 5(a–c), the comparisons for |*a*_*n*_| between analytical results from equation (18) and FDTD results from equation (22) are illustrated with *l*_*pv*_ = 2, 2,25 and 2.5 (corresponds to case A, B and C, respectively). The FDTD results of |*a*_*n*_| have been normalized to the root of summation of their squares. Once again, the two results match quite well.

The actual mean OAM charge of fractional PVs is also calculated, which is shown as dots in Fig. 5(d) and the analytical predictions by equation (14) are also plotted as a solid line for comparison. Although there is a little divergence due to calculation errors in FDTD simulation, the numerical results are consistent with the analytical prediction of equation (20) so that our proposed method of sculpturing fractional PVs by utilizing radial phase gradient of incident LG beam is verified.

## Discussion

Above all, the incident LG beams are constrained with radial index number *p* = 0. If higher radial index number *p* ≥ 0 is considered, the associated Laguerre polynomial could be alternatively positive and negative with increasing radius. In addition, it is much harder to ensure the intensity balance of LG beam along the ASG as the main and side lobes of LG beam are getting closer with increasing *p*. As a result, excited PVs could not be probably expressed with a simple analytical expression as equation (19) anymore. However, a more flexible method of controlling PVs could be expected in turn. This is a far more interesting topic needs to be further explored.

Besides, an updated ASG^35^ is suggested here to further reduce the amplitude variation along the groove. Such segmented but shorter ASG would bear less intensity variation from the incident LG beam, as the fractional contribution is also segmented into several parts. The less the fractional contribution, the smaller the radius variation of ASG. Though tiny differences exist between the two kinds of ASGs, the basic principle is the same. Further work on this topic would be carried out later.

In this paper, fractional PVs are theoretically analyzed by employing the Fourier expansion. The method of utilizing radial phase gradient induced by incident LG beam propagation is proposed to sculpture PVs from integer to fractional OAM. The preset OAM of an excited PV could include four parts: incident SAM, OAM, the geometric contribution of chiral plasmonic structure and the radial phase gradient contribution of incident LG beam. As the beam parameters can be dynamically varied with a commercialized SLM, our proposal provides a continuous method for sculpturing PVs with either integer or fractional OAM.

## Method

In the FDTD numerical simulations, a LG beam (freespace wavelength *λ* ~ 633 nm) with designed parameter shown in Table 1 are set as the incident, towards a closely located gold membrane (thickness ~200 nm) with ASG structures. The boundary condition is set as perfect matched layer. Contrast with the analytical derivations above, there are no assumptions in the simulation.

## Additional Information

**How to cite this article**: Wang, Y. *et al*. Dynamically sculpturing plasmonic vortices: from integer to fractional orbital angular momentum. *Sci. Rep.* **6**, 36269; doi: 10.1038/srep36269 (2016).

**Publisher’s note:** Springer Nature remains neutral with regard to jurisdictional claims in published maps and institutional affiliations.

## Figures and Tables

**Table 1:** The beam parameters of five cases shown in Fig. 3.

Case	Optical waist/μm	Propagation distance/μm	Fractional number
A	*w*(0) = 8	*z* = 0	*α* = 0
B	*w*(0) = 3.75	*z* = 120	*α* = 0.25
C	*w*(0) = 2.25	*z* = 70	*α* = 0.5
D	*w*(0) = 1.28	*z* = 51	*α* = 0.75
E	*w*(0) = 1	*z* = 38	*α* = 1
